# Initial allocation of flood drainage rights based on a PSR model and entropy-based matter-element theory in the Sunan Canal, China

**DOI:** 10.1371/journal.pone.0233570

**Published:** 2020-06-01

**Authors:** Fuhua Sun, Xiuping Lai, Juqin Shen, Libing Nie, Xin Gao

**Affiliations:** 1 College of Agricultural Science Engineering, Hohai University, Nanjing, Jiangsu Province, China; 2 Business School, Hohai University, Nanjing, Jiangsu Province, China; 3 Economics and Management School, Wuhan University, Wuhan, Hubei Province, China; The University of Hong Kong, HONG KONG

## Abstract

The pursuit of flood prevention safety and the mitigation of drainage contradiction against an unnecessary influx of floodwater require a modern and efficient model to optimize the management of the initial allocation of flood drainage rights. We attempted to formulate a framework for initial flood drainage rights allocation to promote the sustainable drainage of the Sunan Canal, China. The Pressure-State-Response (PSR) model was constructed using a literature review and interviews with experts and directors using 18 key indicators being determined from field surveys and library studies. We then assessed the flood status of Zhenjiang City, Changzhou City, Wuxi City and Suzhou City in the Sunan Canal zone using an entropy-based matter-element model. The flood drainage rights for a total of 400m^3^/s was allocated to the four cities in accordance with their flood status. Our research demonstrated that, overall, the four cities may gain the flood drainage rights of 106.67m^3^/s,120.40m^3^/s, 118.22m^3^/s and 54.71m^3^/s, respectively. Specifically, the calculation of the flood drainage for Wuxi was very close to the actual allocation in 2016, whereas there were differences in the other cities that should not be neglected.

## Introduction

Since ancient times, China has been a country with frequent floods. There has been an increasing level of investment in the construction of flood control and drainage systems, including more than 85,000 reservoirs of various sizes, a total of 286,900 km of levees of various standards, and 98 flood detention zones with a total area of 34,500 km^2^ and a total volume close to 100 billion m^3^ [1]. These investments have contributed substantially to flood control in China. However, satisfactory flood protection has not been achieved even with massive efforts and powerful embankments [2]. The country continues to be plagued by hazardous floods, and existing structures neither keep destructive waters away from the people nor distance the later away from the floods [3], particularly in the context of global warming and human activity [4]. During the period from 2010–2018, according to data collected by the Ministry of Water Resources of the People’s Republic of China, disastrous floods caused direct economic losses of 239.04 billion yuan, and thousands of people in this densely populated country were affected [5].

Traditional drainage requires draining water into rivers with the least possible delay [6]. However, the increasing proportion of impermeable surfaces intensifies runoff and places multiple intense pressures on drainage systems [7], which heightens the vulnerability of cities to waterlogging and inundation [8]. Thus, many regions have been valuating advanced flood control criteria and drainage capacity[9]. During seasons of excessive rainfall, such as the Mei-yu season in the Yangtze valley, regions overwhelm the capacity of the river channel as they are motivated to divert water and are equipped with drainage capability [10]. Not unexpectedly, this may result in some negative consequences such as crowding and frequent flood disasters [11]. Coordination and cooperation are at the center of resolving the inevitable conflicts of interest among different regions [7]. At present, regional coordination primarily depends on flood control. Most provinces, cities and even some large basins have executed flood control plans, including the Yangtze valley, Taihu Basin, Haihe Basin, etc. However, flood control remains a global problem and needs further improvement [12, 13], particularly in the field of non-structural measures. Compared with structural measures, non-structural measures focus on the scientific implementations of flood protection demand and are necessary supplements to structural measures [14]. The allocation of flood drainage rights, which is one supplementary non-structural measures, is thus conducive to sustainable flood prevention and mitigation.

Flood drainage rights refer to the government-owned right to discharge floodwater under the constraint of the flood-carrying capacity and with the premise of flood control safety and the requirement of social equity [15]. The management of these rights aims to reduce runoff progressively through the reasonable allocation of floodwater capacity resources. The principle of drainage management is widely recommended and applied in many developed countries, although the terminology varies [6]. In Europe, Sustainable Urban Drainage Systems (SUDS) are increasingly considered an appropriate strategy to control the excessive flow of stormwater [16, 17]. The U.S. Environmental Protection Agency (EPA) has developed stormwater drainage plans and related regulations with the guidance of the National Pollutant Discharge Elimination System (NPDES) [18]. To achieve zero growth of water discharge, Germany encourages every household to adopt the technology of rainwater harvesting, storage and utilization through drainage costs with the amount close to sewer fee [19]. Over time, China has established a relatively complete detention and drainage system through the development of drainage projects such as drainage ditches, pumps, gates, etc. This system provides a practical foundation for the allocation of flood drainage rights based on some limited, preliminary studies. Of these studies, only a few discuss how to allocate flood drainage rights. Yu et al. explored the concepts and influencing factors of regional allocation based on a case study of the Huaihe River [20, 21]. Zhang et al. constructed a bi-level multi-objective programming model and improved the allocation result from the perspective of fairness and efficiency [9], and Shen et al. presented a chaos optimization projection pursuit with reference to the allocation of scarce resources [22]. Despite these distinct bodies of work exploring different allocation methods, few studies related to flood drainage rights have simultaneously considered the different factors of society, economy, environment and flood control safety in an attempt to disentangle the true contribution of each to flood control.

Before we apply flood drainage rights to mitigate flood control pressure, it is necessary to evaluate the flood risk status of an area as the basis for scientific, representative and feasible allocation. The "Pressure-State-Response" model (PSR model), which was developed to meet the challenge of urgent environmental issues [23], provides a powerful tool to separate the causality of many impact factors [24, 25]. It is an effective method for the assessment of ecosystem health and security [26, 27], coastal vulnerability [28], ecotourism carrying capacity [29], etc. Given that flood risk status assessment is a complicated decision-making process with multiple indicators [15], the PSR model is often combined with other assessment methods such as matter-element extension [30–32]. Matter-element extension theory was introduced by Cai in 1983 [33] and studies the elements of a matter [34]. As the factors of pressure, state and response are simultaneously affected by one another, it is appropriate to use matter-element theory to evaluate flood risk status because of its efficiency in solving in non-compatible problems [35].

This paper aims to introduce an allocation model of flood drainage rights in areas potentially prone to pluvial flooding. In section 2, the study area is reviewed briefly and variables are selected for the PSR framework. The entropy method is adopted to calculate weights. The proportion allocated to each region was then determined based on the flood status that was determined using the matter-element extension method. The calculations and analysis are demonstrated for the studied area in section 3. Finally, brief conclusions are drawn in section 4.

## Materials and methods

### Study area

The Grand Canal is the world's longest man-made waterway, and the section in Jiangsu Province is known as the Sunan Canal. This section lies entirely in southern Jiangsu Province, China. From its source at Jianbi in Zhenjiang City, the canal flows from North to South across the Taihu Lake Basin, collecting the waters of its major tributaries, down to Yaziba on the border of Jiangsu Province and Zhejiang Province. The Sunan Canal has a total length of approximately 210 km with 42.6 km in Zhenjiang City, 44.5 km in Changzhou City, 41.4 km in Wuxi City and 81.7 km in Suzhou City.

Historically, the Grand Canal was designed to meet the demands of shipping. It also provides water drainage for nearby areas during the flood season. However, with the industrialization and urbanization that has accompanied rapid development, the canal has been become the main channel for the discharge of floodwater from the cities along its length. Since 2003, Jiangsu Province has proceeded with a channel regulation project that transforms the original Grade Ⅳ channel into a Grade Ⅲ channel, which alters the water regime. Some higher areas such as Huxi, which is west of Taihu Lake, previously discharged floodwater through the canal to the Yangtze River and now drains water to the south. This project places little emphasis on controlling the floodwaters of the canal. The cities along the canal, however, including the cities of Suzhou, Wuxi and Changzhou, have already constructed flood protection encirclements and have raised the flood protection standard in central areas to one event every two centuries. Some low-lying polder areas are not far behind. Now, the total flow discharged into the canal reaches 1048m^3^/s, which is well above the safe flow of 400 m^3^/s that was estimated by the Jiangsu Provincial Department of Water Resources in June, 2016. These changes place considerable pressure on flood control in the Sunan Canal.

In critical situation, the canal acts as a special "reservoir" for water regulation and storage during intense drainage of floodwater from the cities. Thus, the drainage contradictions are increasingly prominent and have become the focus of the Taihu Lake Area’s Flood Control and Drought Relief Headquarters, particularly in 2016, when these areas were struck by several long-lasting heavy storms related to the super El Nino phenomenon during the end of June and early July. In addition, this was the first year since the completion and initial operation of the flood protection project encirclements in the cities. As proposed by the Jiangsu Provincial Department of Water Resources, flood drainage rights allocation is a compulsory measure to address this dilemma.

### Methods

#### PSR model

**Fig 1:**
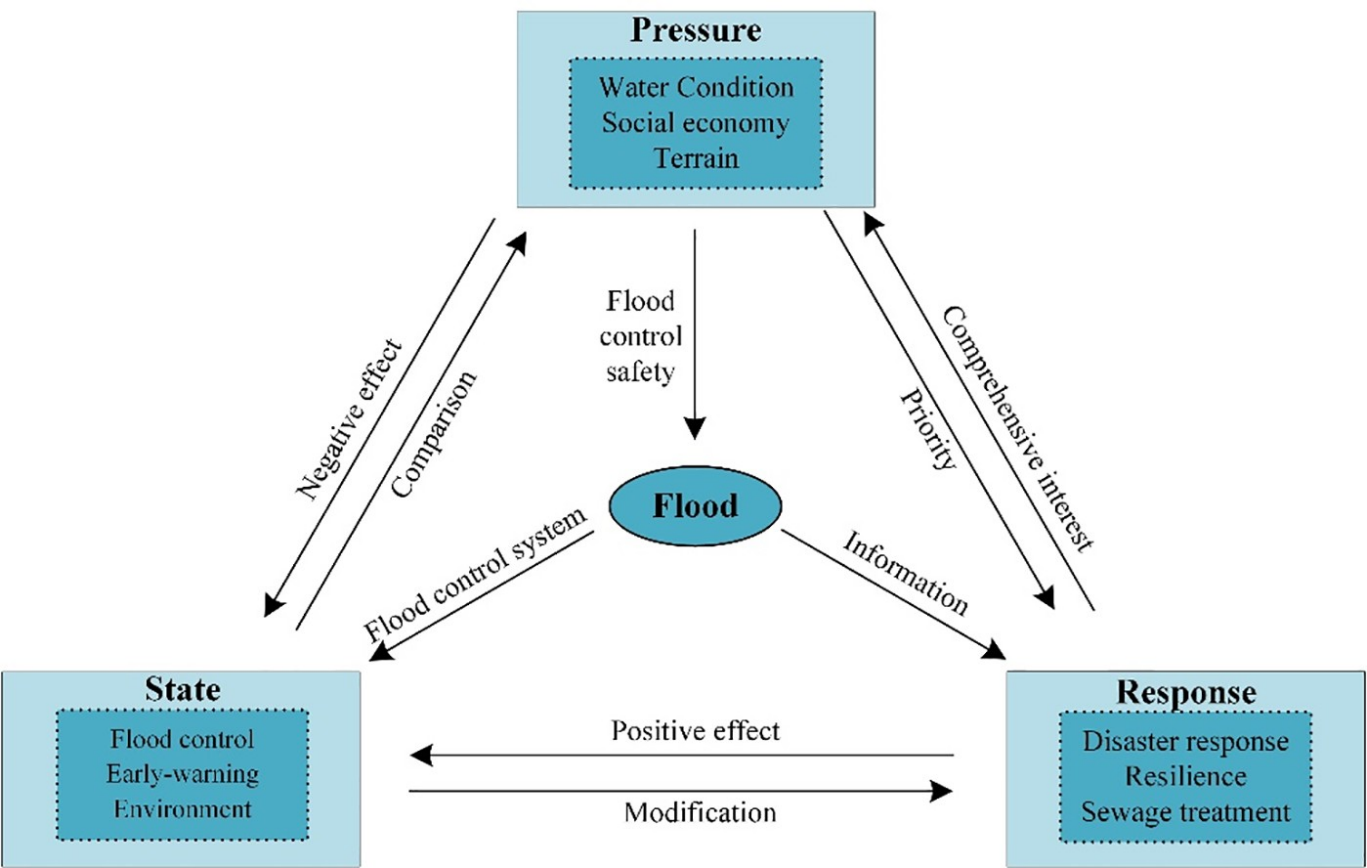
PSR model of initial allocation of flood drainage rights.

The PSR model proposed by David J. Rapport and Tony Friend in 1979 consists of three elements: pressure, state and response. This is a conceptual framework associated with the causality of what has occurred (pressure), the current status (state), and what action should be taken (response). The PSR model of initial allocation of flood drainage rights includes three parts: a) the pressure related to water, the social economy, terrain etc.; b) the status of flood control, capacity for early-warning and environmental conditions; c) the capacity for response such as anti-disaster response, resilience and sewage treatment (see Fig 1).

Interviews with experts and literature reviews together with the principles of quantification, accessibility and spatial variability were utilized to select the pressure, state and response indicators. (see Table 1).

**Table 1:** Indicators for the initial allocation of flood drainage rights.

Factors	Index	Explanations
Pressure	Rainfall intensity(year) ***(p***_***1***_***+)***	Frequency of maximum fifteen-day rainfall
	Water level (%) ***(p***_***2***_***+)***	The degree to which the water level surpasses the warning level
	Population density(people/km^2^) ***(p***_***3***_***+)***	people per km^2^
	GDP (10^4^ RMB) ***(p***_***4***_***+)***	GDP per capita
	Watershed shape coefficient ***(p***_***5***_***+)***	Ratio of watershed border to the circumference of a circle with the same area
	Effective drainage area(km^2^) (***p***_***6***_***+***)	The total catchment area of the Sunan Canal for each region
State	Fixed assets investment in water conservancy facilities (%) ***(s***_***1***_***-)***	Ratio of fixed assets investment in water conservancy facilities to GDP
	Water area (%) ***(s***_***2***_***-)***	Proportional water area
	Total reservoir storage capacity (10^8^ m^3^) ***(s***_***3***_***-)***	Total storage capacity of all reservoirs
	Levee length(km) ***(s***_***4***_***-)***	Total length of levees
	Hydrological sites ***(s***_***5***_***-)***	The number of hydrological sites
	COD emission(kg/10^4^rmb) ***(s***_***6***_***-)***	The scale of COD emission
Response	Drainage scale(m^3^/s) ***(r***_***1***_***-)***	Maximum drainage scale
	Capacity for command and control ***(r***_***2***_***-)***	Staff in flood control headquarters
	Capacity to handle emergent events ***(r***_***3***_***-)***	Emergency support of materials, transportation, communication, rescuing project, etc.
	Average number of people per worker needs to support ***(r***_***4***_***+)***	From the statistical yearbook
	Inequality coefficient of spatial income distribution (%) ***(r***_***5***_***-)***	Ratio of per capita rural net income to per capita urban disposable income
	Daily sewage treatment capacity (10^4^ t) ***(r***_***6***_***+)***	Daily scale of sewage treatment

"+" represents a positive indicator, "-" represents a negative indicator.

Table 1 shows that the pressure project layer includes three aspects: water conditions, social economic vulnerability and terrain. The water regime includes two indicators: rainfall intensity and water level. The variability in the frequency and intensity of precipitation has a significant influence on flooding events[36]. In regards to social economy, population density reflects the ability to transfer people and the increase in social problems when flood events occur, and GDP per capita reflects the development level of each region[37]; both of these parameters are influenced by floodwater[38]and they comprehensively represent the vulnerability of the social economy. The terrain factor is described by two indicators: the watershed shape coefficient and the effective drainage area, which reflect the influence of land surface conditions on the hazards of a flood event.

The state project layer includes three factors: flood control capacity, early-warning capability and the ecological environment. Flood control capacity is embodied by fixed asset investment in water conservancy facilities, total reservoir storage capacity, water area and the length of levees. The larger the water area, the larger the capacity for flood regulation in the region. Water conservancy facilities, reservoir storage capacity and levees were used to characterize the capacity for human intervention in a flood. Additionally, the number of hydrological sites represents the early-warning capacity of each region. Furthermore, as there are too many contradictions caused by water disqualifications when charging floodwater, it is essential to consider the floodwater quality, which is constrained by COD emissions. Flood protection should be approached in an environmentally sustainable manner and should not introduce unwarranted disturbances to the ecosystem [39].

The response project layer represents the types of measures that reduce the negative impact from the pressure, including disaster response capability, resilience and sewage treatment capacity. Among these factors, disaster response capability is represented by the ability and resources to handle floods such as drainage scales, capacity for command and control, handling emergent events, etc. Resilience is represented by the average number of people per worker needs to support and the inequality coefficient of spatial distribution. Sewage treatment capacity is represented by the daily sewage treatment capacity, which promotes the construction of the ecological civilization emphasized in Xi Jinping's report at the 19th CPC National Congress.

#### Entropy method

There are two general methods used to assign index weights: subjective and objective. Subjective judgement is highly dependent on the knowledgeability of the experts you involved and is fraught with uncertainty, gaps in experience and uncertain accuracy. The objective method relies more on the indicators themselves and utilizes mathematical models or theory. The entropy method is derived from Shannon [40] who combined entropy with information. Shannon suggested that entropy could be used to quantify how much information the data could provide and to evaluate the uncertainty of the information. This technique evolved into an evaluation method to determine weights using the degree of variability in the data. The lower the Shannon entropy, the more information the indicators can provide [41]. A unified system for evaluating the initial allocation of flood drainage rights is not available at present. It is necessary to rely on the subjective judgment of experts in the process of determining indicators, and therefore it is easy to produce unreasonable results by introducing personal bias in the process of assigning weights. Therefore, the subjectivity and human influence can be reduced by introducing the entropy method to address the issues of order, degree and utility [42]. The steps to determine the weights were as follows[43, 44].

*(1) Normalization*. To eliminate the dimensionality and magnitude of different indicators so that they can be compared in time and space, the indices need to be standardized in the range between 0 and 1. Suppose that the numbers of evaluation objects and evaluation indicators are m and n, respectively; in the evaluation system, the original judgement matrix is formed:

$$X={\left(x_{ij}\right)}_{m\times n}$$ (1)

where *x*_*ij*_ is the original value of the *i*_th_ indicator of the *j*_th_ object and *i*, *j* = 1, 2, …, n.

The indicators are divided into positive indicators and negative indicators[45]. For positive indicators, a larger value is better, whereas for a negative indicator, the opposite is true. The parameter *a*_*ij*_ is the standardized value *a*_*ij*_∈[0,1]. The positive indicators are standardized by

$$a_{ij}=\frac{x_{ij}-\min_{1\leq i\leq m}x_{ij}}{\max_{1\leq i\leq m}x_{ij}-\min_{1\leq i\leq m}x_{ij}}$$ (2)

whereas the negative indicators are standardized by

$$a_{ij}=\frac{\max_{1\leq i\leq m}x_{ij}-x_{ij}}{\max_{1\leq i\leq m}x_{ij}-\min_{1\leq i\leq m}x_{ij}}$$ (3)

then the standardized evaluation matrix is formed:

$$A={\left(a_{ij}\right)}_{m\times n}$$

*(2) Entropy weights*. Entropy represents uncertainty. By the definition of entropy, the entropy of the *i*_th_ initial allocation indicator is calculated from the relation:

$$E_{i}=-\frac{1}{\ln m}\sum_{j=1}^{m}f_{ij}∙\ln f_{ij}$$

$$f_{ij}=\frac{a_{ij}}{\sum_{j=1}^{m}a_{ij}}$$ (4)

where 0≤*E*_*i*_≤1, when *f*_*ij*_ = 0, $\lim_{f_{\mathrm{ij}\to 0}}f_{ij}∙\ln f_{ij}=0$.

The entropy weight of the *i*_th_ initial allocation indicator can then be given as:

$$\omega_{i}=\frac{1-E_{i}}{\sum_{i=1}^{n}\left(1-E_{i}\right)}$$ (5)

#### Matter-element extension method

The proportion of flood drainage rights allocated is determined by its weight. The extension of matter-element can be used to confirm the evaluation criteria through qualitative analysis, whereas the degree of correlation can be used objectively to determine the allocation weights through quantitative analysis. Through the combination of quantitative and qualitative methods, the challenges of the conventional method, which is subjective or inaccurate, are resolved. The process of initial flood drainage rights allocation based on the matter-element extension method is as follows: first, the matter-element and its classical domain are constructed; then the joint domain and matter-element to be judged are determined; and then the weight of the initial allocation of flood drainage rights in each region is determined by the calculation of the approach degree [35, 46].

*(1) Determination of matter-element and classical domain*. *For* a given matter, its basic elements can be described with an ordered triple: *R = (N*, *c*, *v)*. In the initial allocation of flood drainage rights, *R* represents the matter-element of the allocation, *N* represents the flood drainage rights to be allocated, *c* represents the indices and *v* represents the measured values of *N* on *c*. It is assumed that there are *m* levels and *n* indicators, and the matter-element matrix of flood drainage right allocation is defined accordingly.

$$R_{0}=\left[\begin{array}{cc} N & \\ c & v \end{array}\right]=\left[\begin{array}{ccccc} & N_{1} & N_{2} & \cdots & N_{m}\\ c_{1} & <a_{11},b_{11}> & <a_{12},b_{12}> & \cdots & <a_{1m},b_{1m}>\\ c_{2} & <a_{21},b_{21}> & <a_{22},b_{22}> & \cdots & <a_{2m},b_{2m}>\\ \vdots & \vdots & \vdots & \ddots & \vdots \\ c_{n} & <a_{n1},b_{n1}> & <a_{n2},b_{n2}> & \cdots & <a_{nm},b_{nm}> \end{array}\right]$$ (6)

where *N*_*j*_(*j* = 1,2,…,*m*) is the *j*_th_ level and *v*_*ij*_ = <*a*_*ij*_,*b*_*ij*_> is the critical threshold of the *i*_th_ indicator corresponding to the level *j*, i.e., the classical domain.

*(2) Determination of joint domain and evaluation of matter-element*. The joint domain is the value range of each index, given by *v*_*ik*_ = <*a*_*ik*_,*b*_*ik*_> (i = 1,2,…,n) and *v*_*ij*_*ϵV*_*ik*_.

$$R_{k}=(N_{k},c,v_{k})=\left[\begin{array}{ccc} N & c_{1} & v_{1k}\\ & c_{2} & v_{2k}\\ & \vdots & \vdots \\ & c_{n} & v_{nk} \end{array}\right]$$ (7)

where *N*_*k*_ is the *k*_th_ region, *c*_*i*_ is the *i*_th_ indicator and *v*_*ik*_ is the value of *c*_*i*_ in *N*_*k*_.

*(3) Calculation of close degree and regional weights*. The final step is the use of the asymmetrical keep close degree law to evaluate the flood drainage rights allocation scale.

$$\rho (v_{ik},v_{ij})=\left|v_{ik}-\frac{1}{2}(a_{ij}+b_{ij})\right|-\frac{1}{2}(b_{ij}-a_{ij})$$ (8)

where ρ(*v*_*ik*_,*v*_*ij*_) represents the distance between *v*_*ik*_ and *v*_*ij*_.

$$K_{kj}(N_{k})=1-\frac{1}{n(n+1)}\sum_{i=1}^{n}\omega_{i}∙\rho (v_{ik},v_{ij})$$ (9)

$${K_{kj}(N_{k})}_{P}=1-\frac{1}{42}\sum_{p=1}^{6}\omega_{p}∙\rho (v_{ik},v_{ij})$$ (10)

$${K_{kj}(N_{k})}_{S}=1-\frac{1}{42}\sum_{s=1}^{6}\omega_{s}∙\rho (v_{ik},v_{ij})$$ (11)

$${K_{kj}(N_{k})}_{R}=1-\frac{1}{42}\sum_{r=1}^{6}\omega_{r}∙\rho (v_{ik},v_{ij})$$ (12)

where *K*_*kj*_(*N*_*k*_) represents the close degree of the *k*_*th*_ region of the *j*_*th*_ evaluation level. The parameters *K*_*kj*_(*N*_*k*_)_*P*_, *K*_*kj*_(*N*_*k*_)_*S*_ and *K*_*kj*_(*N*_*k*_)_*R*_ are the close degrees of the pressure, state and response subsystems, respectively, in the *k*_*th*_ region of the *j*_*th*_ evaluation level.

If the contribution coefficients of the different levels are *θ*_*j*_; the integrated close degree of the *k*_*th*_ region can then be calculated by Eq (13):

$$K(N_{k})=\sum_{j=1}^{m}\theta_{j}∙K_{kj}(N_{k})$$ (13)

$${K(N_{k})}_{P}=\sum_{j=1}^{m}\theta_{j}∙{K_{kj}(N_{k})}_{P}$$ (14)

$${K(N_{k})}_{S}=\sum_{j=1}^{m}\theta_{j}∙{K_{kj}(N_{k})}_{S}$$ (15)

$${K(N_{k})}_{R}=\sum_{j=1}^{m}\theta_{j}∙{K_{kj}(N_{k})}_{R}$$ (16)

where *K*(*N*_*k*_) represents the integrated close degree of the *k*_*th*_ region. The parameters *K*_*kj*_(*N*_*k*_)_*P*_, *K*_*kj*_(*N*_*k*_)_*S*_ and *K*_*kj*_(*N*_*k*_)_*R*_ are the integrated close degrees of the pressure, state and response subsystems, respectively, in *k*_*th*_ region.

The greater the integrated close degree of the *k*_*th*_ region, the greater the demand and the greater the corresponding weight of flood drainage rights. The regional weights of flood drainage right allocations can be calculated by Eq (17):

$$W_{k}=\frac{K(N_{k})}{\sum K(N_{k})}$$ (17)

## Results and discussions

### Index values and weights

There are four cities along the Sunan Canal: Zhenjiang City, Suzhou City, Wuxi City and Changzhou City. After determining the indicators, we used the data for 2016 collected from statistical yearbooks, the Jiangsu Water Resources Yearbook (2016), the Water Resources Department of Jiangsu Province and similar sources to assign values, and the entropy weight method was then used to assign the weights; the results are shown in Table 2. The flood risk in Wuxi and Changzhou is greater than the other cities as they experience intense rainfall more than once a century, particularly Changzhou, where the water level is well above the warning level. Moreover, Suzhou possesses the largest water area and the longest levee, which indicates that its flood control capability is relatively strong, whereas Zhenjiang, the poorest city, has the lowest drainage scale value and the lowest sewage treatment capacity.

**Table 2:** Index values and weights for flood drainage rights allocation in the Sunan Canal.

Index	Zhenjiang	Suzhou	Wuxi	Changzhou	Weights
***p***_***1***_	16	16	119	119	0.0628
***p***_***2***_	18.39	19.82	26.79	40.16	0.0612
***p***_***3***_	828	1230	1411	1077	0.0528
***p***_***4***_	12.06	14.81	14.40	12.49	0.0566
***p***_***5***_	2.80	2.05	2.87	3.19	0.0517
***p***_***6***_	643	665	858	749	0.0588
***s***_***1***_	5.96	1.74	7.95	3.84	0.0536
***s***_***2***_	16.54	36.70	27.81	16.77	0.0529
***s***_***3***_	8.2	0	1.85	7.8	0.0600
***s***_***4***_	1293.32	7418.25	2213.97	2656.95	0.0514
***s***_***5***_	14	33	16	23	0.0522
***s***_***6***_	1.02	0.20	0.10	0.13	0.0512
***r***_***1***_	272	325.60	582.60	499.06	0.0545
***r***_***2***_	4.13	5.87	6.52	8.70	0.0536
***r***_***3***_	6.92	7.69	8.46	10.00	0.0553
***r***_***4***_	1.4	1.58	1.39	1.64	0.0604
***r***_***5***_	50.06	50.96	54.19	51.63	0.0517
***r***_***6***_	57	379	132	120	0.0592

### Flood drainage rights allocation results

There are no universal standards or methods for the grading index. The actual situation of each region must be considered synthetically, and the grading standards should be able to distinguish the differences between different regions. As a result, the priority of flood drainage rights has been divided into three levels with level I being the highest priority, followed by level II and level III. Table 3 provides the detailed criteria for the levels.

**Table 3:** Criteria for assigning priority.

Index	I	II	III
***p***_***1***_	>50	20~50	<20
***p***_***2***_	>30	20~30	<20
***p***_***3***_	>1200	1000~1200	<1000
***p***_***4***_	>14	12.5~14	<12.5
***p***_***5***_	>3	2.5~3	<2.5
***p***_***6***_	>800	700~800	<700
***s***_***1***_	>5	2~5	<2
***s***_***2***_	<20	20~30	>30
***s***_***3***_	<2	2~6	>6
***s***_***4***_	<2000	2000~5000	>5000
***s***_***5***_	>25	15~25	<15
***s***_***6***_	<0.5	0.5~1	>1
***r***_***1***_	<400	400~550	>550
***r***_***2***_	>8	6~8	<6
***r***_***3***_	>8	6~8	<6
***r***_***4***_	>1.6	1.3~1.6	<1.3
***r***_***5***_	>55	50~55	<50
***r***_***6***_	>200	100~200	<100

Eqs (6) and (7) were used to determine the classical and extensional matter elements, and Eqs (8)–(16) were used to calculate the integrated close degree of each region and the integrated close degrees of the pressure, state and response subsystems in each region, respectively. Finally, Eq (17) was used to determine the allocation results on the basis of the calculations above.

$$R_{0}=[\begin{array}{cccc} & N_{1} & N_{2} & N_{3}\\ p_{1} & <50.150> & <20,50> & <0,20>\\ p_{2} & <30,50> & <20,30> & <10,20>\\ p_{3} & <1200,1500> & <1000,1200> & <800,1000>\\ p_{4} & <14,16> & <12.5,14> & <10,12.5>\\ p_{5} & <3,3.5> & <2.5,3> & <2,2.5>\\ p_{6} & <800,900> & <700,800> & <600,700>\\ s_{1} & <5,8> & <2,5> & <1,2>\\ s_{2} & <15,20> & <20,30> & <30,40>\\ s_{3} & <0,2> & <2,6> & <6,9>\\ s_{4} & <1200,2000> & <2000,5000> & <5000,7500>\\ s_{5} & <25,35> & <15,25> & <10,15>\\ s_{6} & <0,0.5> & <0.5,1> & <1,1.5>\\ r_{1} & <250,400> & <400,550> & <550,700>\\ r_{2} & <8,10> & <6,8> & <4,6>\\ r_{3} & <8,10> & <6,8> & <4,6>\\ r_{4} & <1.6,2> & <1.3,1.6> & <1,1.3>\\ r_{5} & <53,55> & <51,53> & <50,51>\\ r_{6} & <200,400> & <100,200> & <50,100> \end{array}]$$

$$R_{k}\left[\begin{array}{ccccc} & v_{1} & v_{2} & v_{3} & v_{4}\\ p_{1} & 16 & 16 & 119 & 119\\ p_{2} & 18.39 & 19.82 & 26.79 & 40.16\\ p_{3} & 828 & 1230 & 1411 & 1077\\ p_{4} & 12.06 & 14.81 & 14.40 & 12.49\\ p_{5} & 2.80 & 2.05 & 2.87 & 3.19\\ p_{6} & 643 & 665 & 858 & 749\\ s_{1} & 5.96 & 1.74 & 7.95 & 3.84\\ s_{2} & 16.54 & 36.70 & 27.81 & 16.77\\ s_{3} & 8.2 & 0 & 1.85 & 7.8\\ s_{4} & 1293.32 & 7418.25 & 2213.97 & 2656.95\\ S_{5} & 14 & 33 & 16 & 23\\ s_{6} & 1.02 & 0.20 & 0.10 & 0.13\\ r_{1} & 272 & 325.6 & 582.6 & 499.06\\ r_{2} & 4.13 & 5.87 & 6.52 & 8.7\\ r_{3} & 6.92 & 7.69 & 8.46 & 10\\ r_{4} & 1.4 & 1.58 & 1.39 & 1.64\\ r_{5} & 50.06 & 50.96 & 54.19 & 51.63\\ r_{6} & 57 & 379 & 132 & 120 \end{array}\right]$$

Using the entropy method, we obtained the index weight coefficients from the calculations above. Because the various levels have different relative importance, the different levels reflect different flood risk. The higher the level, the higher the flood risk. Supposed *θ*_*j*_ = [0.8,0.6,0.2] as in reference [47], the weight coefficient is substituted into Eq (9); the resulting close degree of each region is shown in Table 4. As seen from Table 4, the highest level for Suzhou is level II, indicating that the drainage demand of Suzhou is lower, which is consistent with the description above.

**Table 4:** Close degree of each region.

	K_I_(N_k_)	K_II_(N_k_)	K_III_(N_k_)	K(N_k_)
Zhenjiang	0.7168	0.4969	0.0819	1.2956
Suzhou	0.1372	0.3484	0.1789	0.6645
Wuxi	0.7560	0.5865	0.0933	1.4359
Changzhou	0.6755	0.6692	0.1177	1.4624

**Fig 2:**
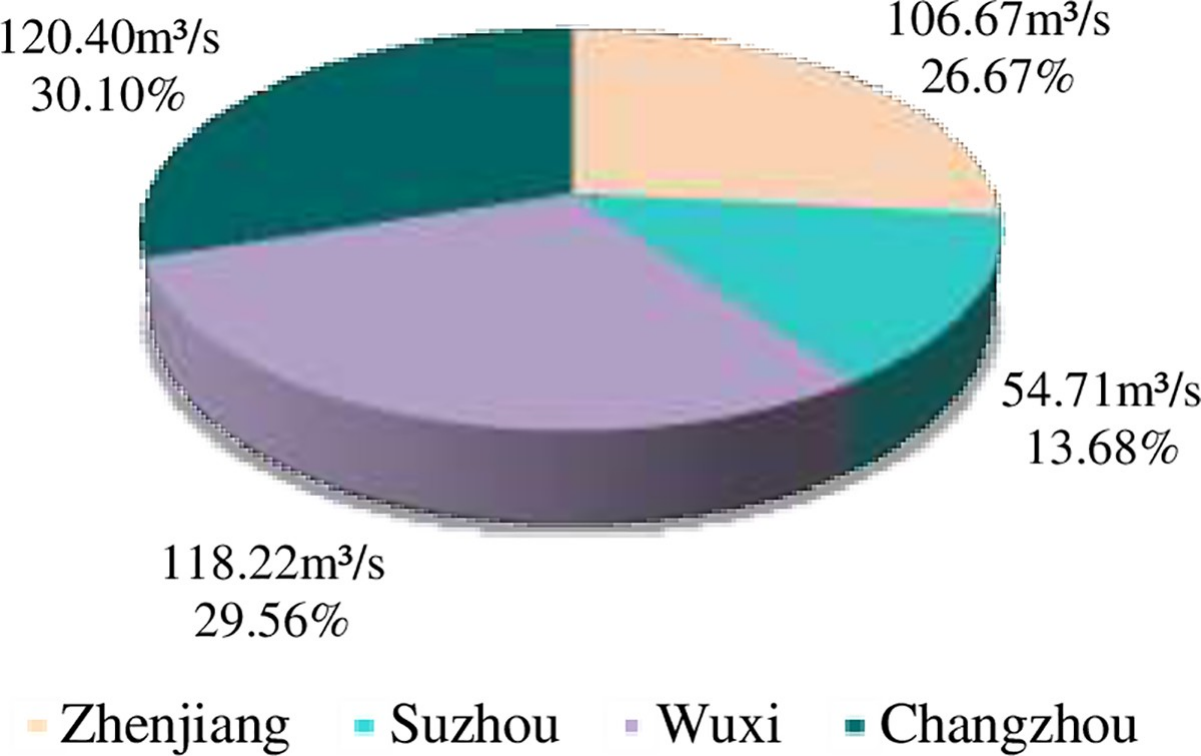
Initial allocation results of flood drainage rights.

**Fig 3:**
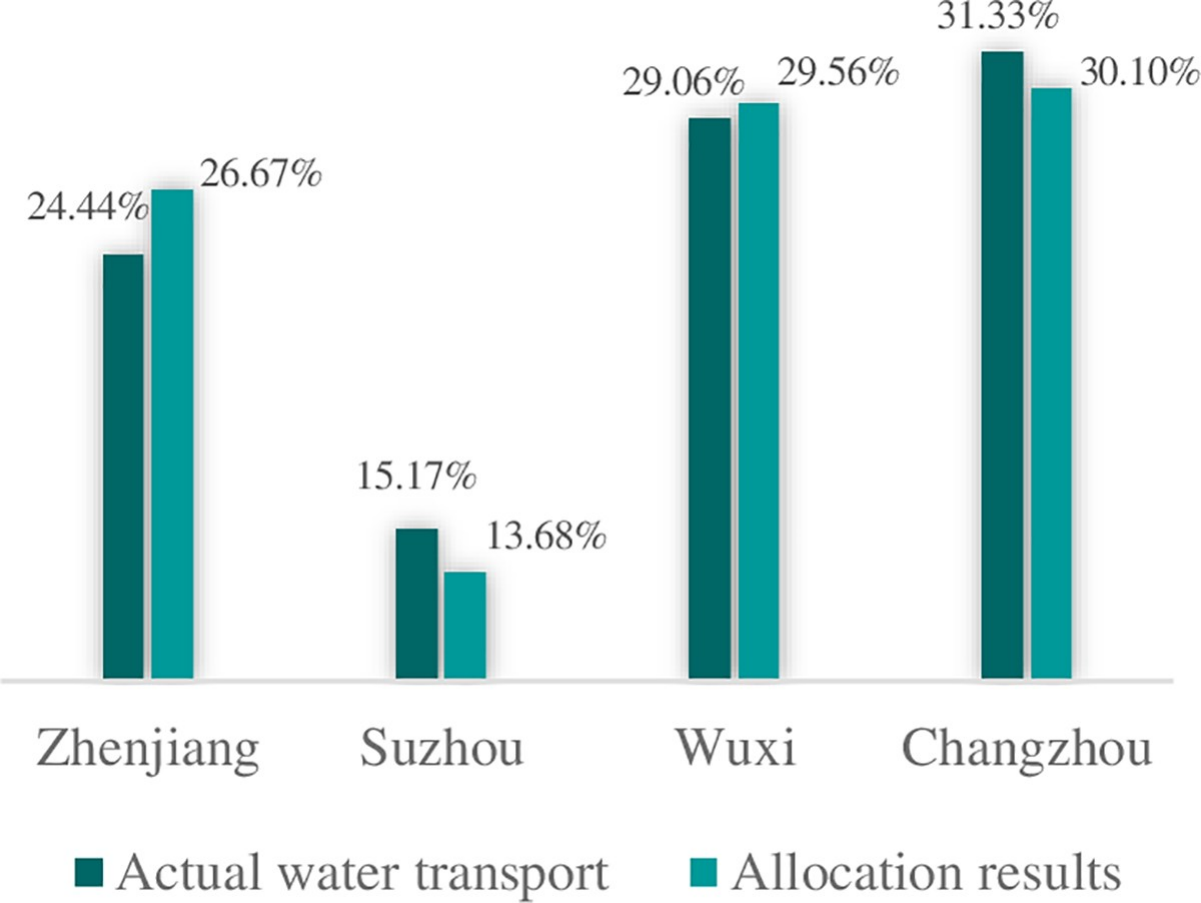
Actual vs calculated results.

After the normalization of the close degree of evaluation for the regions, the proportional flood drainage rights allocation among the four cities as listed in Table 4 were 26.67%, 13.68%, 29.56% and 30.10%, respectively. The total flood drainage rights of the Canal during this period was 400m^3^/s; the allocation of this quantity is shown in Fig 2. Based on the pressure that each city faces, the state and the response capability of each city, the drainage scales of Zhenjiang, Suzhou, Wuxi and Changzhou were 106.67m^3^/s, 54.71m^3^/s, 118.22m^3^/s and 120.40m^3^/s, respectively. Wuxi and Changzhou therefore receive more flood drainage rights and Suzhou receives the fewest rights accordingly. The allocation results were generally consistent with the actual water transport; the water transport of the canal in Zhenjiang, Suzhou, Wuxi and Changzhou were 24.44%, 15.17%, 29.06% and 31.33%, respectively (see Fig 3).

Among the cities, Wuxi City and Changzhou City had the maximum rainfall, which was greater than the once-in-a-century type of rainfall. The water levels of these two cities surpassed the warning level by a greater margin than the other cities, particularly in Changzhou City, where water rose to 6.32 m on July 3, 2016, which is 2.02 m above the warning level. Both of these indicators demonstrate that Changzhou and Wuxi were faced with greater risks of flooding. Suzhou was allocated minimal flood drainage rights of 13.68% due to possessing the longest levee, the largest water area, receiving the least precipitation and exhibiting a relatively low water level.

### Analysis of different cities

**Fig 4:**
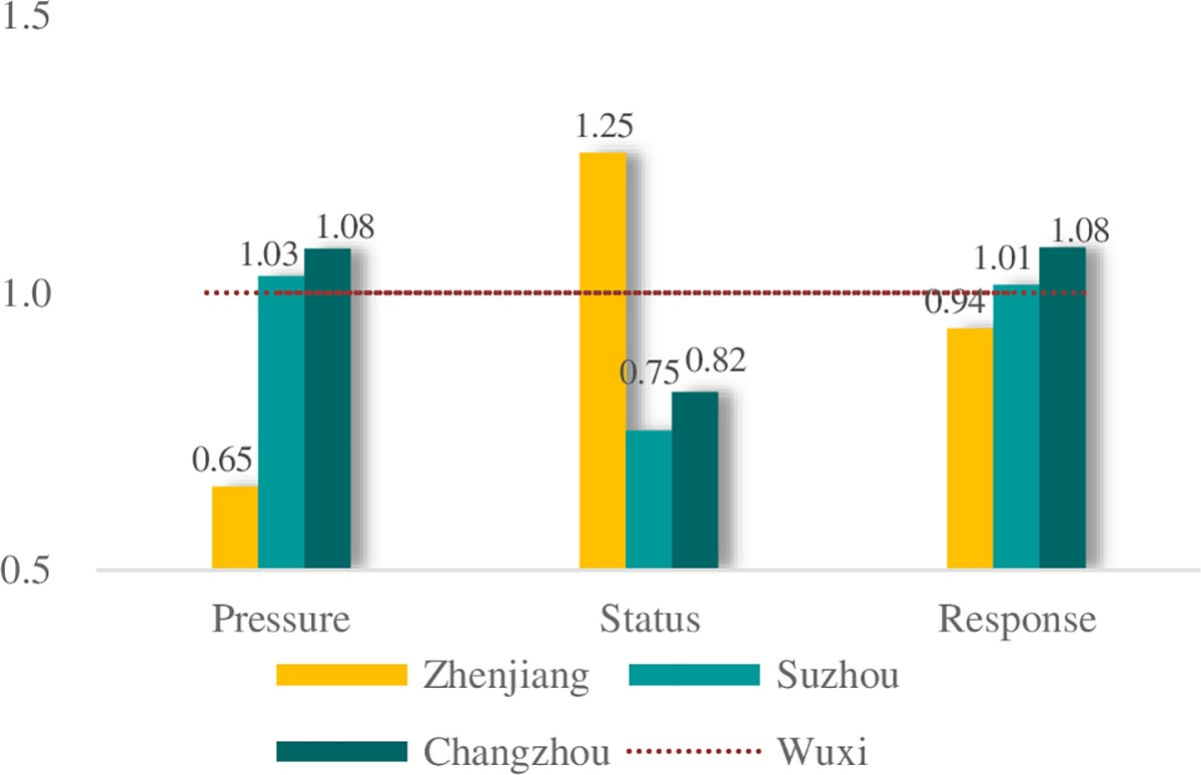
The close degrees of the four cities for the different layers.

The results are similar to, but not completely equivalent to, the actual situation. There was no notable difference between the calculated result and actual result in Wuxi City. However, there were differences of over 1% in the other cities, particularly in Zhenjiang (2.23%). The flood disaster caused a direct economic loss of 513 million yuan in Wuxi, 5.262 billion yuan in Changzhou and 409 million yuan in Zhenjiang, respectively[48]. Among them, both Wuxi and Changzhou had suffered much heavier rainfall and higher water levels. And Wuxi is also the most populous city and has the higher economic development level than Changzhou. It means Wuxi had achieved a desired result as expected. It was plausible to suggest that the actual allocation of Wuxi in 2016 was at a reasonable level. Thus, Wuxi City was designated as the reference city. For Wuxi, all of the close degrees in the pressure, state and response subsystems were set equal to 1. The close degrees of the other cities were all standardized to Wuxi. These results are shown in Fig 4. Among these values, the higher the close degree in the pressure subsystem, the greater the flooding pressure in this region. A higher close degree in the state subsystem indicates that the flood control capability needs considerable attention; the same is true for the response subsystem.

Fig 4 shows that in the response subsystem, the abilities to respond to flood hazards were approximately equal for all the cities. Therefore, we focused our analysis of the cities on the pressure and state subsystems.

#### Zhenjiang

Zhenjiang City, which is located in the upstream section of the Sunan Canal and has a major discharge outlet, the Jianbi pump station, and experienced less flood pressure than Wuxi. The main indicators such as rainfall, water level, population and the drainage area in Zhenjiang were far less than those in Wuxi, as was the GDP per capita. This indicates that the flood risk and socioeconomic development in Zhenjiang are less than in Wuxi. However, the status in Zhenjiang appears to be far above that of Wuxi. The reason, as indicated by the matter-element extension matrix, is that the water conservancy facilities, water area and levee length in Zhenjiang were less than in Wuxi, which means Zhenjiang has less room and flexibility for artificial adjustment when there is a heavy rainfall and the water level in the Canal rises.

#### Suzhou

Suzhou City was allocated the smallest proportion of flood drainage rights because it received the least rainfall and possessed the lowest water level in the pressure subsystem, and it also possessed a good status. Taihu Lake is the diversion and storage center for flood control in the Taihu Basin, the flood control safety of which is related to Jiangsu Province, Zhejiang Province and Anhui Province. The Taipu floodgates and Wangting water conservancy project, both of which are located in Suzhou, are the most important outlets for Taihu Lake. As a result, Suzhou invests more than other cities in the construction of water conservancy projects. Suzhou has the largest water area and the longest levee, which protects water quality by preventing dirty and substandard floodwater from entering Taihu Lake. Consistent with the principle of maintaining the flood control safety of Taihu Lake, Suzhou City was also urged to reduce floodwater discharge.

#### Changzhou

Because Changzhou City showed poor performance in both the pressure subsystem and the state subsystem, it was allocated the largest proportion of flood drainage rights. Statistically, the precipitation from July 1 to 4 in Changzhou City reached an astounding 294.8 mm with the maximum precipitation of 450.0 mm occurring at the Maodong Reservoir in the Jintan District. Consistent with this extreme rainfall, the water levels monitored by the major station along the mainstream, Changzhou Station, continued to rise and reached the highest point of 6.32 m at 9 a.m. on July 5, which is 2.02 m above the warning water level of 4.30 m. This level of water would cause serious waterlogging in Zhonglou District, Wujin District and Xinbei District, which are at elevations that should not have been flooded.

In regards to the state subsystem, Changzhou is less developed than Wuxi with a lower GDP and a relatively lower investment in water conservancy projects. In addition, Changzhou does not have the same water area, a quantifiable indicator of floodwater handling capacity, as Wuxi. However, the reservoir storage capacity of Changzhou is much greater than Wuxi, which vastly improves its status, as does the levee length. Having more hydrological sites indicates that hydrological data collection at Changzhou is more accurate and rapid. The scant margins of indicators ***s***_***1***_**, *s***_***2***_ and ***s***_***6***_, in contrast, appeared less noteworthy. Besides, Changzhou is the upstream of Wuxi and Suzhou. The effect of coupling greater flood pressure and better flood control status meant that Changzhou required only slightly more flood drainage rights than Wuxi, as shown in our results.

## Conclusion

The initial allocation of flood drainage rights is a powerful tool to reduce flood risk and to address the drainage contradictions in the main river during periods of excessive rainfall. The investments in the construction of flood control and drainage systems have been increasing on a large scale but are far from economical. The management role of flood drainage rights is conducive to producing the maximum benefit under the existing waterworks conditions. Flood control is also an integrated complex problem involving multiple driving factors and stakeholders and should be fostered in an equitable, balanced and effective manner.

Before allocating flood drainage rights, the first step is to evaluate the potential for flooding in each region. The factors influencing the allocation of flood drainage rights include water conditions, social economy, terrain, flood control status, early-warning capability, environmental conditions, disaster response capability, resilience capability and sewage treatment capacity. We introduced the PSR model to establish our evaluation index after the analysis of these relationships. Our analysis showed that rainfall, water level, reservoir storage capacity and employment were the most important factors in the weighted results. A comprehensive evaluation index system enhances the rationality, accuracy and scientificity basis of the results. Thus, this paper may lay a foundation for future research on flood drainage rights. Using the matter-element extension theory, an entropy-based matter-element model was adopted to determine the proportional distribution of flood drainage rights among the cities of Zhenjiang, Changzhou, Wuxi and Suzhou in China during 2016. This evaluation enriched the models and methods for the initial allocation of flood drainage rights. Additionally, the feasibility of the model was verified by the comparison and analysis between the calculated and the actual situation.

Our work does not come without limitations. It is necessary to consider location when applying our model. The analysis framework of influencing factors may apply to many areas, but there is a pressing need for different areas with particular circumstances to use unique combinations of indicators. Moreover, we expect that our research could be applied to the allocation of flood drainage rights in areas with more complicated drainage relationships. However, if an area has two or more channels to discharge their floodwater at the same time, the method may need improvement, and the allocation may require a higher level of coordination.

Currently, the research and practice of the initial allocation of flood drainage rights are in their infancy in China. To expand its application, the construction of evaluation indices and allocation models yet trading management are of crucial importance, which needs to be explored further in combination with different local situations. We suggest that studies on the theory and methods initial allocation of flood drainage rights in different regions be conducted in the context of promoting the establishment of property relations for contemporary natural resources. Case studies may play an important role in the popularization and application of flood drainage rights allocation. Pilot work on the management and mechanism of the initial allocation is necessary and would also provide a reference for decisions on the flood control optimization operations.

## Supporting information

S1 Data (XLSX)
